# GC-MS profiling and bioactivity prediction of compounds from *Momordica charantia L.* extract

**DOI:** 10.6026/973206300181009

**Published:** 2022-10-31

**Authors:** Pinak Pani Nath Choudhury, Sudip Choudhury

**Affiliations:** 1 Department of Zoology, S. S. College, Hailakandi, Assam - 788151, India; 2 Center for Soft Matter, Department of Chemistry, Assam University, Silchar, 788011, India

**Keywords:** bitter melon, phytochemical, fruit tissue, ethyl acetate, extract, GCMS

## Abstract

*Momordica* species are vegetable crops, belonging to the family of *Cucurbitaceae*. For the present study plant samples of *Momordica charantia* were collected from different parts of Barak Valley of Assam, India. Hyphenated technique Gas Chromatography-Mass Spectroscopy (GC-MS) of the chromatographically separated components of the plant extract was performed to identify some of the principles. All the isolated compounds exhibited notable interaction with the enzyme Bone morphogenic protein upon *in silico* screening. All the compounds were screened *in silico* for toxicity and then docked with the target enzyme individually. It was found that these compounds are interacting with the target enzyme and is able to inhibit the enzyme activity more efficiently as compared to the reference ligand.

## Background:

The quest for new pharmacologically active agents obtained by screening living sources such as microbial fermentations and plant extracts has led to the discovery of many clinically useful drugs that play an important role in the treatment of human ailments [1]. Momordica charantia is a vegetable crop also known as bitter melon, karela, etc is a medicinal plant from the Cucurbitaceae family; it is predominantly cultivated in Africa, Asia, and South America [2,3]. This plant is a traditional herbal medicine, possesses various pharmacological functions, namely antidiabetic, abortifacient, anthelmintic, contraceptive, antimalarial and laxative. It is used for the treatment of dysmenorrhea, eczema, gout, jaundice, leprosy, piles, pneumonia, psoriasis, rheumatism and scabies [4-6]. Bioactive compounds, phytochemicals include numerous sterols, terpenoids, phenolic compounds, proteins, peptides, amino acids, carbohydrates, fatty acids, flavonoids, vitamins, and metals have already been reported from the plant [7]. A significant hypotensive effect of M. charantia in younger adults and in short-term interventions was reported by Jandari et al. 2020 [8]. Kumar et al. 2009 hypothesize that M. charantia fruit extracts act as insulin sensitizer and activate the glucose transport in a PI3K dependent fashion [9]. M. charantia not only shows hypoglycemic activity but also play an active role in prevention as well as delay in progression of diabetic associated complications in animal models [9-11]. The 80% ethanol–water combination solvent extract of M. charantia exhibited marked antioxidant activities according to both the DPPH and FRAP assays [12]. The green fruits and leaves of Momordica species play a major role in improving human health by offering nutritional and nutraceutical components [13]. Soo May L et al. 2018 reported Momordica charantia supplementation can be used as an alternative to reducing pain among the primary knee osteoarthritis patients [14]. Anti‑arthritic activity of ethanolic extract of M. charantia is reported by Kola V et al. 2018 [15]. Insilico docking study of Luteolin, isolated from M. charantia was proved to have good affinity for target protein responsible for Alzheimer's disease [16]. In recent years, it is found that GC-MS analysis is a valuable technique to identify various bioactive constituents, like, esters, alkaloids, steroids, amino compounds, and nitro compounds. Gas chromatography, coupled with identification based on the mass of the separated compounds, makes GC-MS an ideal technique for the qualitative and quantitative analysis of volatile and semi-volatile phytochemicals using small quantities of samples [17, 18]. Again, there has been increasing trends of using in silico methods like reverse screening and network pharmacology in natural products research, because these are well-suited for studying the multi-targeted action of medicinal plants [19]. From the literature survey, it is evident that, much work has been carried out on methanolic and ethanolic extract of M. charantia but no work is reported on M. charantia, growing in Barak Valley region of Assam. The present study aims to study the insilico bioactivity of compounds isolated from ethyl acetate extract of Momordica charantia L. Fruit collected from Barak valley region of Assam using column chromatography, Gas chromatography and mass spectroscopic techniques.

## Materials:

### Plant samples:

Plant samples of Momordica charantia grows in different parts of Barak Valley during whole year especially during the rainy season. The species was identified as Momordica charantia after consulting the herbarium sheet, preserved at Department of Life Science, Assam University, Silchar, India. Fruit of the plant were collected from several growing areas in Barak Valley during Nov - Dec, 2016. The plant material was cut into small pieces and air dried at ambient temperature (∼25°C) and then powdered. The powdered material was used for further experimentations.

### Equipment:

GC-MS analysis was carried out in a combined PerkinElmer Clarus 680 gas chromatograph system and Clarus 600C mass spectrophotometer, with the Acquisition Parameters: Oven: Initial temp 60°C for 3 min, ramp 6°C/min to 200°C, hold 3 min, ramp 6°C/min to 300°C, hold 10 min, InjA auto=280°C,Volume=0 µL, Split=10:1, Carrier Gas=He, Solvent Delay=10.00 min, Transfer Temp=200°C, Source Temp=180°C, Scan: 40 to 600Da, Column 60.0m x 250µm.

### Computational details:

The docking studies were done with AutoDOCK v4.2.6 software package [20]. The molecular structures of the ligands used in the docking studies were generated using Avgedro [21], and online Java applet provided by MolSoft [22]. All the ligand structures were optimized at MM2 level (single molecule, gas phase) before docking. Necessary format conversions of the molecular structure files were done using Open Babel 2.3.0 [23] and Mol2Mol [24]. The FAF-Drugs ADMET tool was used to screen the ADMET profile of isolated compounds [25]. PharmMapper platform was used to identify the probable target for the ligand molecules [26].

## Results:

### Solvent extraction of Momordica charantia:

The extraction of the desiccated and grinded aerial parts (fruits) of Momordica charantia in large scale was performed exhaustively by soxhlet apparatus. The material was first defatted by extraction with petroleum ether. The defatted plant material was then extracted with ethyl acetate. The ethyl acetate extract was dried in vacuo using rotary evaporator to get the crude extracts (Ex2). The processing of extraction is following these steps as shown in the Figure 1(see PDF). The ethyl acetate extract, Ex2 was suspended in hexane. After filtration and concentration, the soluble phase was fractionized by column chromatography on a 2.5x50 cm column over activated silica gel (100-200 mesh) using a gradient of petroleum ether : ethyl acetate as eluent (petroleum ether : EtOAc = 98:2). One fraction was collected on the basis of TLC analysis. On drying in vacuo, this fraction gave yellow substances (ET1) shown in Figure 2(see PDF).

### GC-MS profiling of ET1:

The refined fraction obtained from column chromatography (ET1) was subjected to GC-MS profiling. Gas Chromatography resolved the material to be a mixture of compounds. The Mass spectral data obtained from GC-MS analysis was compared with the mass spectral library to identify each compound from the mixture. From the Gas chromatogram, Figure 3(see PDF) it is clear that the isolated fraction contains a mixture of compounds. The gas chromatogram provides picks at different retention time for the fraction isolated from ethyl acetate extract coded as ET 1. An extensive library search using NIST mass spectral library was done to identify the compound from the library. From the library search, it was found that the compounds isolated from the plant were fatty esters. Phyto components detected in ET 1 using GCMS are given the Table 2(see PDF).

### ADMET screening of the isolated compounds and Identification of probable drug target:

ADMET is an acronym in pharmacokinetics and pharmacology for absorption, distribution, metabolism, excretion, and toxicity. The FAF-Drugs ADMET tool was used to screen the ADMET profile of isolated compounds. The parameters used for the screening are as follows (Table 3 - see PDF). The ADME/Tox property of all the compounds (see Table 4 - see PDF) provide by Mobyle@rpbs server [25] is presented in Table 4(see PDF). It was found that the compounds possess diverse molecular property and some of them may withstand with the drug safety profile. From the table it is clear that the server accepted all the 5 compounds for in silico drug safety analysis but out of 5 compounds, it is found that only 3 compounds are having good solubility, whereas the others are having reduced solubility.

### Identification of probable drug target:

Identification of the probable drug target(s) for an active natural principle is of utmost importance in Bioinformatics studies. For this purpose, PharmMapper server [26] was used to identify the probable target molecules based on the pharmacophore profile of the molecule submitted. The structure of the compounds isolated was converted to sdf format using the software openbabel software [23] and each of the sdf file was uploaded to the pharmapper server [26] for drug target identification. The pharmmapper server [26] provided the target enzyme for each of the compounds. It is found that almost all the compounds are having affinity to bind with Bone morphogenetic protein 2, (PDB ID: 1REU) [28]. Hence, this particular enzyme was taken as target enzyme for docking study with all the isolated compounds. The Bone morphogenetic protein-2 (BMP-2) (Figure 4 - see PDF; PDB ID: 1REU) and members of the TGF-be ta super family regulate the maintenance, development & regeneration of tissues and organs. [28]. BMP-2 has important roles during embryonic development, as well as bone remodeling and homeostasis in adulthood [29].

### Study of binding efficacy of isolated compounds and receptor through docking:

In this study, AutoDock 4.2 [20] was used to screen the binding mode of the isolated compound with the drug target identified from the PharmMapper server [26] result. This molecular mechanics-based approach attempts to predict the structure of the intermolecular complex formed between two or more constituent molecules. This tool may be used to predict the strength of association or binding affinity between two molecules using scoring functions. Docking is frequently used to predict the binding orientation of small molecule, drug candidates, to their protein targets in order to predict the affinity and activity of the small molecule. Hence docking plays an important role in the rational design of drugs. As per the Autodock brochure, the Gibbs free energy of binding of the ligand to the target is calculated as follows:

P(aq) + L(aq) _ PL(aq)

Kb = [PL]/[P].[L]; binding constant

Kd = [P].[L]/[PL]; dissociating constant

Gbinding = -RT.ln Kb = RT.ln Kd

where,

P is Protein or target molecule

L is Ligand or small molecule

G binding is binding Gibb's free energy

R is a constant and T is absolute temperature

### Results of docking study:

The 3D structure of the target enzyme Bone morphogenetic protein 2, PDB ID 1REU was downloaded from https://www.rcsb.org/. The reference ligand, water molecule etc. were removed from the pdb structure using Molegrow molecular viewer. The protein so prepared was loaded in MGL Tools software and was prepared for docking using standard procedure of the software. All calculations for protein-fixed ligand-flexible docking were done using the Lamarckian Genetic Algorithm (LGA) method. The docking site on protein target was defined by establishing a grid box with the dimensions of X: 78 Å Y: 126 Å Z: 126 Å, with a grid spacing of 0.375 Å, centered on X: -18.367 offset -0.333; Y: -20.019 offset 1.861; Z: -39.191 Å offset 1.833. The best conformation was chosen with the lowest docked energy, after the docking search was completed. Ten runs with AutoDock were performed in all cases per each ligand structure. The result obtained is given below in Table 5(see PDF) for each compound. Only the best two pose were saved considering the minimum binding energy and cluster RMSD value.

In Table 5(see PDF), we have listed only the first and second ranked docking pose having the minimum RMSD value i,e. 0.00 from the AutoDock result. It is evident from the table that all the 5 compounds isolated from ethyl acetate extract of Momordica charantia shows good binding efficacy with the target enzyme as compared to the reference ligand. Some of the isolated compound even shows better binding affinity towards the target enzyme.

From the MGL tools, it is found that the Hydrogen atom no11 and 4 of the reference ligand molecule is having affinity to hydrogen bond with CYS47 and CYS79 residue of the target enzyme while the isolated compound_5 is also having the same affinity with a more efficient mean binding energy in the same active site of the enzyme. The Ligplot view of the ligand protein interaction & pose view of the docking pattern of the two molecules viz the reference ligand and the identified compound_5 with the target enzyme is given below in Figure 5(a), 5(b) and Figure 6(a) 6(b)(see PDF).

## Discussion:

This study reveals that the column fraction of ethyl acetate extract of Momordica charantia fruits growing in Barak valley region contains at least 5 methyl esters. Out of these 5 compounds 8-nonynoic acid, methyl ester, butane, 2,2-dimethyl and sulfurous acid, octyl 2-propyl ester, showed good solubility and oral bioavailability. In silico drug target identification showed that all these compounds are having affinity towards the Bone morphogenetic protein-2 (BMP-2) (PDB ID: 1REU). From the in silico docking study, it is found that the compound sulfurous acid, octyl 2-propyl ester, show better mean binding energy as compared to the reference ligand. Moreover the 2nd ranked pose of the reference ligand and 1st ranked pose of sulfurous acid, octyl 2-propyl ester, binds with the target protein in the same active site and interact with the same amino acids residue CYS47 and CYS79 of the target protein through hydrogen bonding, where the isolated compound sulfurous acid, octyl 2-propyl ester, showed better Mean Binding Energy with same RMSD value 0.00.

## Conclusions:

From the literature survey it was evident that Momordica charantia supplementation can be used as an alternative to reducing pain among the primary knee osteoarthritis patients. Present study also supports that the ethyl acetate extract of Momordica charantia contains some of the active principle to support the claim. It should be noted that extensive clinical study is required to establish the same.
